# Possibilities of a Personal Laser Scanning System for Forest Mapping and Ecosystem Services

**DOI:** 10.3390/s140101228

**Published:** 2014-01-10

**Authors:** Xinlian Liang, Antero Kukko, Harri Kaartinen, Juha Hyyppä, Xiaowei Yu, Anttoni Jaakkola, Yunsheng Wang

**Affiliations:** 1 Department of Remote Sensing and Photogrammetry, Finnish Geodetic Institute, Masala 02431, Finland; E-Mails: antero.kukko@fgi.fi (A.K.); harri.kaartinen@fgi.fi (H.K.); juha.hyyppa@fgi.fi (J.H.); yu.xiaowei@fgi.fi (X.Y.); anttoni.jaakkola@fgi.fi (A.J.); 2 Department of Geography and Geology, University of Turku, Turku 20014, Finland; E-Mail: yunsheng.wang@utu.fi

**Keywords:** forest, ecosystem service, laser scanning, LIDAR, point cloud, personal laser scanning, PLS, wearable

## Abstract

A professional-quality, personal laser scanning (PLS) system for collecting tree attributes was demonstrated in this paper. The applied system, which is wearable by human operators, consists of a multi-constellation navigation system and an ultra-high-speed phase-shift laser scanner mounted on a rigid baseplate and consisting of a single sensor block. A multipass-corridor-mapping method was developed to process PLS data and a 2,000 m^2^ forest plot was utilized in the test. The tree stem detection accuracy was 82.6%; the root mean square error (RMSE) of the estimates of tree diameter at breast height (DBH) was 5.06 cm; the RMSE of the estimates of tree location was 0.38 m. The relative RMSE of the DBH estimates was 14.63%. The results showed, for the first time, the potential of the PLS system in mapping large forest plots. Further research on mapping accuracy in various forest conditions, data correction methods and multi-sensoral positioning techniques is needed. The utilization of this system in different applications, such as harvester operations, should also be explored. In addition to collecting tree-level and plot-level data for forest inventory, other possible applications of PLS for forest ecosystem services include mapping of canopy gaps, measuring leaf area index of large areas, documenting and visualizing forest routes feasible for recreation, hiking and berry and mushroom picking.

## 1. Introduction

Laser scanning, also known as LIDAR, is a surveying technique for collecting a three-dimensional (3-D) point cloud of the reflected objects that uses laser ranging and detection, scanning, positioning and orientation measurement techniques. Several types of laser scanning systems currently exist, such as: airborne laser scanning (ALS), terrestrial laser scanning (TLS) and mobile laser scanning (MLS). Many laser scanning systems are multi-sensoral platforms, and therefore the technical development of sensors and advances in computing technology allow new development possibilities.

Forest resource management is one of the main driving forces in the adoption of laser scanning. The first commercial ALS prototype dedicated to topographic mapping was introduced in 1993. Shortly afterwards, ALS was utilized in forest investigations [1–4]. The rapid development of sensors expedites the research and acceptance of the technique. ALS first became semi-operational for inventorying forest in the early 2000s and was used operationally in the late 2000s in Nordic countries. The application of ALS in forest system investigations largely depends on the quality and quantity of field reference data, especially with the currently applied ALS-based inventory technique, which is an area-based inventory where field reference determines the output of each raster cell based on non-parametric estimates [5].

Reference data for forest inventories are conventionally collected by utilizing expensive and labor-intensive manual measurements [6,7]. Both destructive and non-destructive methods can be applied in field data collection. Destructive measurements are typically accurate but highly expensive and are not applicable or acceptable in many cases, such as in urban forests and in conservation areas. Non-destructive measurements are typically carried out by utilizing simple tools, such as calipers and measuring tape. Tree attributes that can be collected are limited to those measurable at reasonable cost and accuracy.

Important tree attributes needed in forest mapping and ecosystem services include the diameter at breast height (DBH), location, tree height, tree species, leaf area index (LAI), the height of the first living branch, stem curve, stem volume and biomass. Many attributes, such as the stem curve, are not practically measurable using simple tools. Allometric models can be used to determine attributes that are not directly measurable but can be derived from other basic measurements. The applicability of allometric functions, however, is limited to particular climatic, geographic and silvicultural conditions. The use of inappropriate allometric models can lead to large estimate errors, as was shown previously [8]. In general, more automated and cost-effective techniques are needed to provide tree attribute data for forest ecosystem services, especially for forest inventories.

Recently, TLS has been proven to be a promising technique in forest field measurements. The first commercial TLS system was built by Cyra Technologies (acquired by Leica in 2001) in 1998, and the first papers related to plot-level tree attribute estimation were reported in early 2000s [9–15]. Currently, TLS has shown to be feasible for collecting basic tree attributes at plot-level, such as DBH and tree position [16–21]. By reconstructing tree stem, it is possible to derive high-quality stem volume and biomass estimates comparable in accuracy with the best national allometric models [22,23].

New TLS systems are continuously being developed, with the size and weight of the laser scanners decreasing rapidly. Dual-wavelength TLS is currently being studied within forest ecology [24]. The use of dual wavelength laser pulses makes it easy to separate leaf returns from returns of stems, branches and the ground, which is convenient for forest ecosystem services. It is expected that TLS will be operationally used in plot-level forest inventories as soon as the appropriate software becomes available, best practices become known and general knowledge of these findings is more widely spread.

Three measurement approaches have been reported in TLS field measurements: single-scan, multi-scan and multi-single-scan. In the single-scan approach, the laser scanner is placed at the center of the plot and one full field-of-view (e.g., 360° in horizontal direction and 310° in vertical direction) scan is made. This approach has the simplest measurement setting and fastest measurement speed in the three approaches because only one scan is applied to a plot. The major problem of this approach, however, is the low detection rate. In the sample plot, 10%–32% of all trees are not scanned from the plot center because of occlusion effects [17,18,20,25].

Several scanning positions are necessary to measure all trees in a plot. In the multi-scan approach, several scans are made inside and outside of the plot. Individual data sets are merged, typically using artificial targets, to form a single point cloud. This approach provides the best data set as the merged point cloud records trees from different directions; however, the approach is not always practical due to the cost of the manual or semi-automated processing required for the registration of several scans. In the multi-single-scan approach, several point clouds are processed individually and data sets are merged at the feature and decision levels. In this approach, the work load is clearly lower than with the multi-scan approach because reference targets are not required and the merging of several scans is fully automated. The detection rate is also clearly higher than that of the single-scan approach because the plot is scanned from several stations.

In practice, convenient measurement methods and rapid data acquisition are always preferred. New possibilities are currently being studied to improve the efficiency of field data collection. Laser scanning has recently been put on moving platforms to build MLS systems and is being studied for forest mapping applications. The main advantage of applying MLS for forest measurements lies in its rapid data collection. Within an equal time frame, the area that can be investigated by utilizing MLS is significantly larger than the area investigated with TLS.

The MLS system consists of one or several laser scanner(s) and multi-sensor positioning and orientation sensors. The first commercial MLS system for surveying applications was StreetMapper, which appeared in the market in 2006. Similar sensor configurations are also used in robotics. MLS systems utilized in surveying and robotics have different emphases and perspectives. Surveying MLS emphasizes an absolute coordinate system and high measurement accuracy. In robotics, relative positions and accuracy are important. Because of the different applications, real-time processing is necessary for robotics but is only an advantage for surveying MLS.

The concept of applying MLS for forest measurement started in early 2010 [26] with the utilization of high pulse/point repetition frequency scanners. When MLS is utilized in stop-and-go mode, similar point cloud data to that collected by TLS are obtained. In such scenarios, multi-sensor positioning and orientation sensors can be used to directly register several scans into a single point cloud, even without the use of separate calibration targets. In the continuous mode, MLS collects similar data to that of ALS. The area covered in the same time span is greater than with the stop-and-go mode, but the produced data set is sparser.

The data collected by MLS systems is less precise in comparison with TLS because positioning errors propagate in the MLS point cloud. Another challenge of applying MLS is that the mobility of the platform in forest environments may be limited. Forest ground is characterized by rugged terrain and obstacles, such as rocks, dead wood and undergrowth. The ground condition may be not easy or even suitable for vehicle movement. In a pilot study, the applied platform was an all-terrain vehicle (ATV) [27].

Personal laser scanning (PLS) is an emerging concept [28]. The idea first appeared as a backpack-type MLS system, where the scanning and positioning systems were on the operator's back rather than on a vehicle platform. The first system prototype was large in size and weighed approximately 30 kg, which limited its operability [29]. It was used in open areas for geomorphological terrain modeling and urban area mapping [29–31]. The last five years have witnessed rapid progress in sensor miniaturization. A new system has been developed with new scanner and multi-constellation GNSS-based navigation systems and was built at the Finnish Geodetic Institute (FGI) in 2013. The new PLS system weighs approximately 10 kg, making it a wearable laser scanning system.

The main advantage of PLS lies in its high mobility in various terrain conditions and rapid data collection. Through professional-quality scanning and navigation systems, the collected data can document objects in detail with high precision. PLS has the potential to improve mapping efficiency compared with conventional field measurements and compensate for the limitations of other laser scanning techniques, such as having to transport the scanner and associated equipment from site to site, which is one major disadvantage of TLS, and having to have certain terrain conditions, which limits the application of MLS. In addition, the PLS measurement scenarios enhance the system capability to record all the trees in the plot because the scanning position and geometry changes all the time. These characteristics are very attractive for forest mapping and ecosystem services.

The objective of this paper is to conduct the first experiment that explores the possibility of applying PLS in forested areas, to demonstrate the first professional-quality PLS for collecting tree attributes for forest mapping and to discuss the implications of utilizing such a system for ecosystem services in a wider context.

## 2. The PLS System, Study Area and Data Acquisition

### 2.1. The PLS System

This study was conducted utilizing the wearable PLS system developed and built at the FGI. The name of the system is AKHKA R2. The system consists of a multi-constellation navigation system, which includes a Global Navigation Satellite System (GNSS) receiver (NovAtel Flexpak6), an inertial measurement unit (IMU) system (NovAtel UIMU-LCI) and an ultra-high-speed phase-shift laser scanner (FARO Focus3D 120). The receiver tracks NAVSTAR's Global Positioning System (NAVSTAR-GPS, abbreviated to GPS) satellites and Global Navigation Satellite System (GLONASS) satellites. Tables 1 and 2 list the technical specification of the navigation system and laser scanner in the PLS.

**Figure 1.**
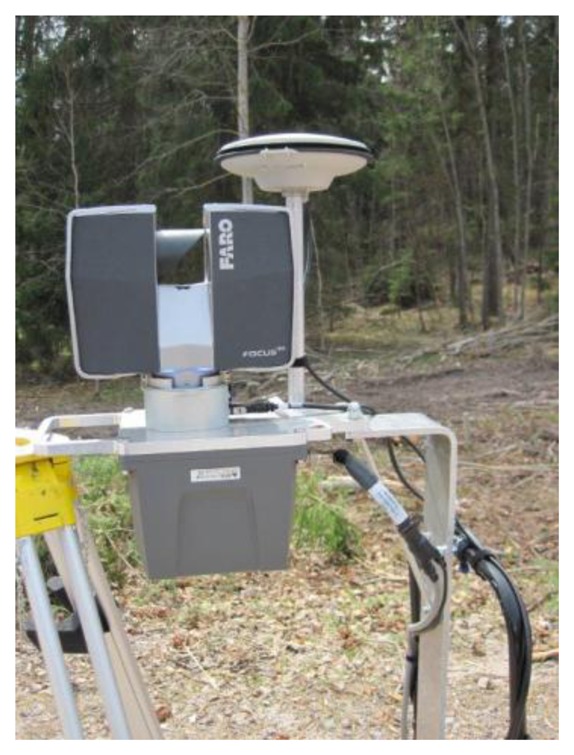
Sensors of the AKHKA R2 system.

In the PLS, the sensors are mounted on a rigid platform. The scanner is installed upwards on the platform with a helical mount kit, the IMU is installed right below the scanner and the GNSS antenna is mounted upwards next to the scanner. The scanner, IMU and GNSS antenna form a single sensor block. The sensor block can also be turned upside down for certain applications, such as ground mapping. The configuration minimizes the need for system calibration because the offsets and rotations between the scanner and IMU remain the same all the time. Figure 1 displays the system configuration.

In operation, the system is on the operator's back and is tilted forward slightly, typically at approximately 20°, and the scanning plane is cross-track. The tilted scanning allows detection of vertical break-lines of stems. If the system was precisely vertically configured, the accuracy of the system for the vertical break-line detection of stems is determined by the scan frequency and walking speed. With the tilted configuration, a higher sampling frequency for break-line features can be achieved because angular resolution within a scan line is significantly higher in comparison with the resolution between scan lines. During the field measurement, swinging up to several degrees can be expected because of the operator's body movement and the system not being tightly fixed on the operator's back.

The GNSS receiver records the platform positions at 1 Hz and the IMU records the platform accelerations at 200 Hz. These data are typically combined in post-processing with reference GNSS data from either a virtual reference data service or physical station on a bench mark to produce the mapping path. The navigation system also records the time signals sent by the scanner. This time signal is utilized to match laser points to scanner position and orientation along the mapping path. 3-D georeferenced points are calculated utilizing the individual scanner positions, the synchronized scanning data and the system calibration data (*i.e.*, sensor offsets and rotations).

### 2.2. The Test Area

The test was carried out in a mature forest plot in an area behind FGI in Masala (60.15°N, 24.53°E), southern Finland. A 2,000 m^2^ (40 m × 50 m) rectangular forest plot was utilized in this study. The dominant tree species in the test area is Scots pine (*Pinus sylvestris L.*). Other species growing in the plot include Norway spruce (*Picea abies L.*), birch (*Betula sp. L.*) and aspen (*Populus tremula L.*). The DBH ranges from 10 cm to 51 cm, and the standard deviation (std. dev.) is 11.48 cm. The tree density is 0.023 stems/m^2^ (DBH over 10 cm). Descriptive statistics of the test plot at the time of the field inventory are summarized in Table 3.

The forest stand structure is built by two layers: an upper story of sparse, large crowns, and a last story of denser shrubs and young coniferous and deciduous trees. The ground is mostly covered by moss and shrubs, and bare bedrock areas and boulders are common. The ground slope of the study area is approximately 9° from north to south. No permanent walking path is present in the test plot.

### 2.3. Data Acquisition

PLS data were acquired in May 2013. The point repetition frequency was 488 kHz, and the scanning frequency was 97 Hz. Virtual reference station (VRS) data and positioning measurements were used in post-processing to compute the mapping path.

**Figure 2.**
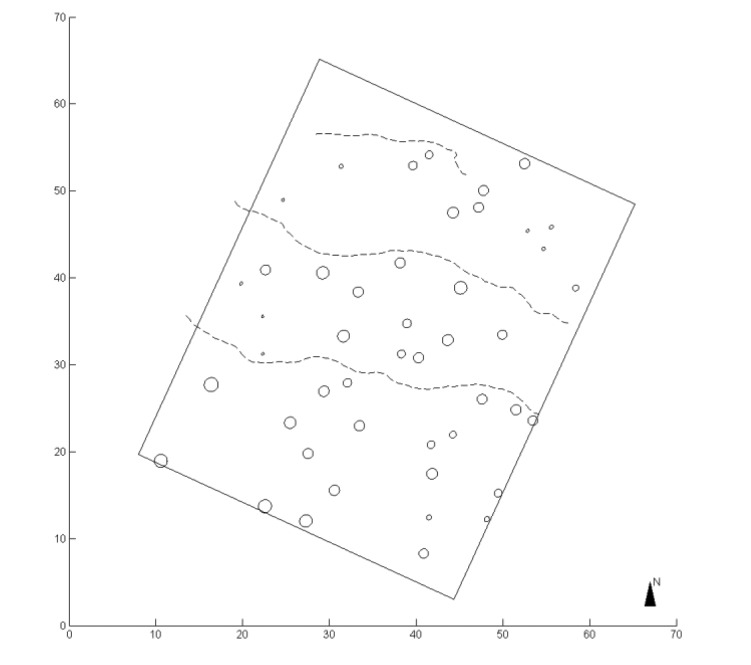
The test forest plot and the mapping paths in the data collection. The rectangular area presents the test plot boundary. The circles and their diameters show the tree locations and corresponding DBHs. The mapping paths are indicated by dashed lines.

Figure 2 displays the test forest plot and the mapping path. The test plot boundary is illustrated as rectangular. The circles and their diameters show tree locations and corresponding DBHs. The dashed lines indicate the mapping path through the plot. In this experiment, the plot was mapped in three walking paths, and the mapping was finished in less than 2 min.

Noise is commonly present in PLS-generated laser point clouds when a phase-based distance measuring system is utilized. Noise points are a result of either weak returns or multiple hits within the laser beam (which yields to a phase slip in the ranging procedure). The scanner employed by PLS provides some on-the-fly filters to diminish noise, such as contour and clear sky filters. This function was turned on in the experiment.

**Figure 3.**
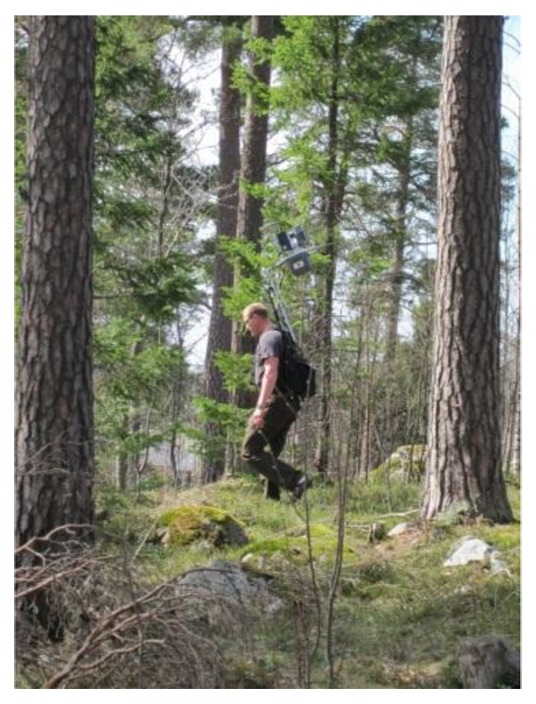
PLS measurement.

**Figure 4.**
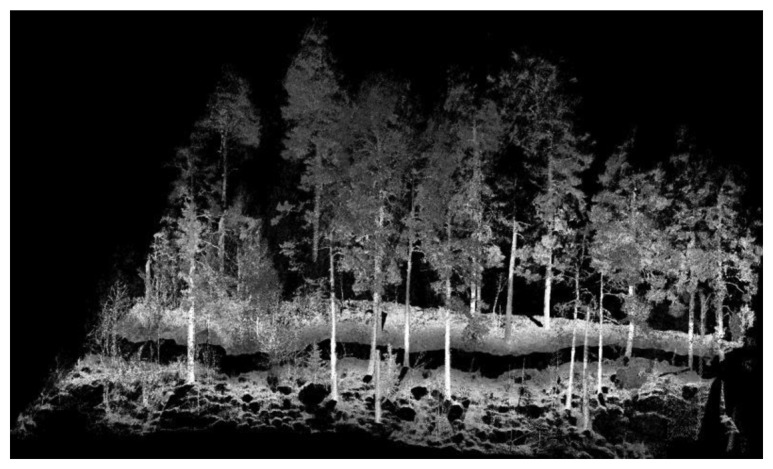
Point cloud data in the test plot utilizing PLS.

The PLS field measurement is shown in Figure 3. As shown in the figure, both dominating and young trees growth in the test area, and bedrocks and shrubs are commonly present. Figure 4 displays a close view of PLS data from the test plot. Points were displayed in shades of grey scale based on the intensity value recorded by the scanner.

The reference data were collected in August 2013. Tree locations were measured from a fixed location inside the plot using a Trimble 5602 DR 200+ total station. The total station was oriented to the same coordinate system as PLS data using VRS-GNSS measurements. The observation location was selected so that trees in the plot are visible. The stem perimeter of each tree was measured by a steel tape to the nearest millimeter at DBH height (1.3 m vertical above the ground from the base of the tree). The DBH was later calculated from the stem perimeter (assuming the tree cross-section is circular) with millimeter accuracy. The tree species were also recorded in the field measurements.

## 3. Methods

### 3.1. Pre-Processing

The raw point data were filtered for noise removal in two phases. The first step deleted noise by intensity and range thresholds and was performed simultaneously with the georeferencing of the point data. Points were excluded if they were beyond a range of *x* meters from the scanner and had an intensity value less than *y*.

In forested areas, visibility is typically poor for stems standing far away from the scanner because of occlusion effects [25]. Taking into account the size of the test plot and the mapping path, 50 m is a value large enough to include meaningful data, so *x* was set to 50 in this experiment. The intensity range of the scanner was 0–2,047. Points with a low intensity value typically have no reasonable accuracy. In this experiment, *y* was set to 700. The threshold of intensity value was experimentally determined.

The second phase was the removal of isolated points. Points were labeled as noise if there were no other points within a 10-cm radius. These points were most likely not reflected from real objects in the surrounding environment. In addition, 100 points were required to be within a 1-m radius to detect small, yet isolated point clusters, which are typically present in phase-shift ranging data. The reduction rates in this phase were approximately 10% of input data.

### 3.2. Mapping Forest Plot

In general, there are two possible scenarios for PLS data processing. The first one is based on co-registration of along-track and across-track data strips by having joint surface patches between the data strips. This approach is similar to the strip adjustment approach applied in ALS [32,33] and the multi-scan approach of the TLS [10,11]. Because of the data acquisition geometry and possible inaccuracies in the GNSS signal under forest canopies, detection of matching patches along and across the strips is more complicated for MLS data than for ALS data processing. A multipass-corridor-mapping approach is proposed in this study, which first processes each data strip individually and then merges the results at the decision level. The first step is similar to the corridor-mapping utilized in ALS applications for power line and highway monitoring. The extracted knowledge of trees is further combined in the second step to map the plot. In general, this approach is similar to the multi-single-scan approach applied in TLS processing.

A corridor was established beside the mapping path, which included points within *d* meters from the path. In this study, *d* is equal to 20. Tree stems within this corridor were detected and then modeled from the laser point data. Each laser point was automatically processed in its neighborhood to identify possible stem points. The size of the neighboring space was determined by the local point density in the neighborhood. The higher the local point density, the more details are recorded in the point cloud; therefore, a smaller size of neighboring space is required to understand the characteristics of objects and preserve the details. When the local point density is low, the features are usually poorly described and a larger neighboring space is necessary to acquire enough neighboring points for the stem detection. The neighboring space is defined by *k*-nearest points, so that the size of the neighboring spaces can be self-adjusted according to the local point density. The *k* value was 100 in this study based on the overall point density evaluation in the whole dataset.

**Figure 5.**
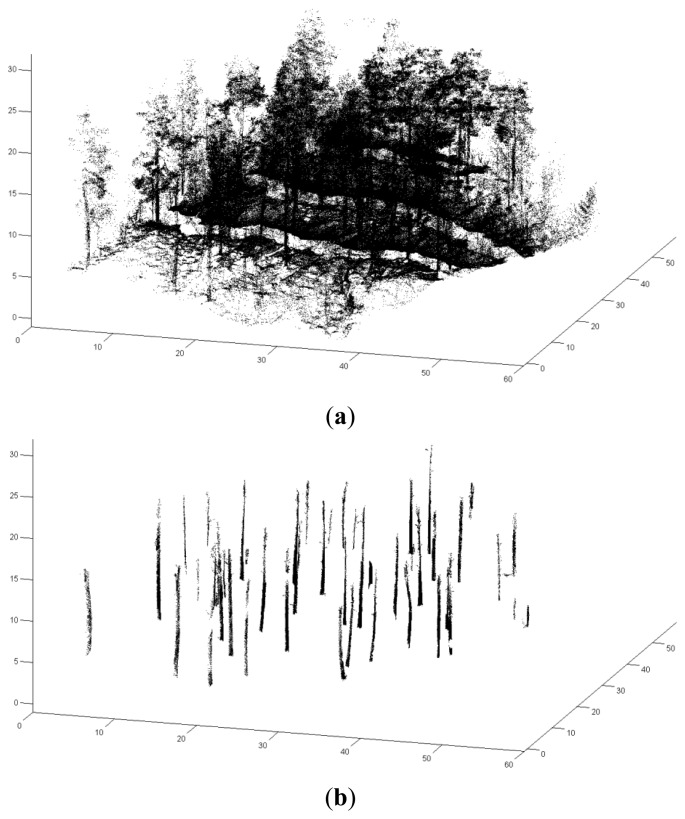
(**a**) Point cloud captured by the PLS system (**b**) and the recognized stem points.

A local coordinate system was established in this space, where eigenvectors indicated the axis directions and eigenvalues indicated the variances of the points along the axes. The planar surface was identified if neighboring points were mainly distributed along two axes in the local coordinate system. The vertical shape was recognized by the normal vector to the surface, which was approximately horizontal in the real world coordinate system. Figure 5a shows the laser points in the plot captured by the PLS and 5b displays the recognized stem points.

Tree stem models were built from the recognized stem points. A series of 3-D cylinders were utilized to represent stem shapes, such as changing growth directions. To match a cylinder to a stem section, laser points were weighed to reduce the influence of noise, such as branch points. For additional details on the modeling procedure, reader is referred to [25]. The DBH and location of the stem were then estimated from the cylinder element at breast height (1.3 m above the ground) and the preliminary map was made based on the stem locations and DBH estimates.

Depending on the path and the size of the buffer, overlaps may exist between corridors. Therefore, a tree may be mapped several times in the preliminary mapping. These results were then analyzed and merged at a decision level.

The merging criteria were the stem location and detected stem points. Several tree stems with the same location were identified as the same tree recorded in different mapping paths. For each detected stem, the laser points were located and the stem with the largest number of points was selected as the final detection. The decision criterion assumes that the more laser points reflected from the stem, the more reliable of a stem model could be made. More discussion on this decision criterion is provided in Section 5. The merged results produced the final plot map.

For comparison, all laser points within the plot were merged as one data set by using navigation signals. This new data corresponds to the multi-scan data in the conventional TLS measurement and was processed utilizing the same detection and modeling procedures as in the individual corridors.

### 3.3. Evaluation Criteria of Mapping Results

The mapping results were evaluated utilizing measured references. The mapping accuracy was evaluated on the basis of omission errors, commission errors and overall accuracy. Omission errors are references that were not mapped. Commission errors are objects that were mapped but did not have corresponding references. Overall accuracy is the percentage of correct detections.

The accuracy of the DBH and position estimates were evaluated utilizing the bias and root mean squared error (RMSE), and the relative bias and RMSE, as defined in Equations (1)–(4):

(1) $$\text{Bias}=\frac{1}{n}\sum_{i=1}^{n}e_{i}=\frac{1}{n}\sum_{i=1}^{n}(y_{i}-y_{ri})$$

(2) $$\text{RMSE}=\sqrt{\frac{\sum {\left(y_{i}-y_{ri}\right)}^{2}}{n}}$$

(3) $$\text{Bias}\%=\frac{\text{Bias}}{{\bar{y}}_{r}}\times 100\%$$

(4) $$\text{RMSE}\%=\frac{\text{RMSE}}{{\bar{y}}_{r}}\times 100\%$$

where *y_i_* is the *i*th estimate, *y_ri_* is the *i*th reference, *ȳ_r_* is the mean of the reference values, and *n* is the number of estimates. The multipass-corridor-mapping method was also evaluated by comparing its mapping results with results obtained from the merged point cloud.

## 4. Results

### 4.1. Multipass-Corridor-Mapping

The results of the stem mapping utilizing the multipass-corridor-mapping method and the PLS system are reported in Table 4. The overall stem-mapping accuracy was 82.6%.

The omission errors were eight reference trees that were not mapped, which included two trees not covered in point cloud data because of shadowing effects, one young spruce tree whose stem was heavily occluded by its branches, and five cases where the stems were barely recorded in the data set.

The commission errors included eleven detected trees without corresponding reference data. One tree was just on the plot border, which was not in the reference because of its location. Ten commissions were tree groups or small trees with a PLS estimated DBH between 10 and 17 cm. The studied forest plot has many saplings, and the references have a DBH greater than 10 cm. In the field measurement, small saplings with a DBH just above 10 cm may not have been recorded because the field crew estimated that they did not equal or exceed the minimum diameter criteria. For the PLS stem modeling, small stems with a DBH just under the minimum criteria may be modeled as a bit larger, so that they were estimated as those fulfilling the diameter criteria. This type of commission error is typical in forest areas where there are many small saplings. The accuracies of the DBH and the position estimates are reported in Table 5.

### 4.2. Merged Point Cloud Mapping

Table 6 reports the results of DBH and position estimates by utilizing the merged point cloud data for comparison. The overall stem-mapping accuracy was 73.9% for this merged data set.

In this case, the omission errors were twelve reference trees that were not mapped, which included two trees not covered in point cloud data because of shadowing effects, one young spruce tree whose stems were heavily occluded by its branches, three trees where the stems were barely recorded in the data set, and six other trees that were not modeled mostly because of mismatching effects.

The commission errors were ten detected trees without corresponding reference data. Two trees are on the plot border, and eight commissions were small trees or tree groups.

The accuracy of the DBH and the tree stem position estimates utilizing merged point cloud are reported in Table 7.

## 5. Discussion

One of the major advantages of applying PLS is rapid data acquisition of the entire forest plot. In this test, it took less than 2 min to map the test plot. The current PLS system is capable of recording approximately 1 million points per second and weighs approximately 10 kg. In the coming years, it is expected that the scanning frequency will be higher and the weight of the PLS systems could be reduced, e.g., to a few kilograms. In principle, the PLS system is easy to put on and operate, and the whole plot can be recorded in a minute or in a few minutes.

The PLS has some clear advantages over TLS when taking field measurements. PLS solves the TLS-related problems of setting up the system and using calibration targets on tripods. In addition, transferring the scanner between scanning positions and from one plot to the next can be faster using the PLS system because all equipment is on the operator's back. Transferring from one plot to the next can be also accomplished using ATVs, which expedites field measurements and makes PLS easy to operate in the field.

In addition, the PLS point cloud is less sensitive to distance than the TLS data. The PLS provides a multi-view mapping scenario that records an object from different directions as the operator position and viewing geometry are constantly changing and records the entire plot by walking through it. A more equally distributed point cloud can be obtained by utilizing PLS. Access to all trees and collecting a point cloud representing all trees can be more easily guaranteed using PLS because the operator can identify areas that deserve additional attention and scan those locations. The errors related to missing trees with the TLS approach can be minimized by using PLS.

Previous research showed that the single-scan approach misses up to 32% of all trees in the sample plot because of occlusion effects [17,18,20,25]. In this study, less than 5% of the reference trees were not scanned by the PLS system. This result confirms the assumption that the multi-view mapping scenario of a PLS system can provide improved plot coverage and reduce occlusion effect in comparison with the single-scan TLS approach where the observation position is fixed.

This result also indicates that it is important to plan the walking path before the measurement to minimize missed trees. In this experimental data set, the south-east corner was hardly covered because of occlusion effects, which were not expected before the experiment. This result shows that the field crew plays an important role in utilizing PLS system because he/she needs to plan the mapping path carefully or identify areas that deserve additional visits.

In this study, a multipass-corridor-mapping method was developed for processing PLS data in a forested area. The method first processes data along the mapping path independently and then merges the detecting results at a decision level. The merging criterion is the number of stem points in each walking path.

The mapping of forest sample plots at the decision level was first proposed in [34]. In that study, the merging criterion is the distance between detected trees and scanner positions. Criteria in the previous as well as in this study assume that good stem visibility leads to accurate stem models. In general, the further away an object is, the smaller the number of laser beams are reflected by the object in single-scan TLS data, because of the larger beam size and sparser point spacing. However, stems close to the scanner may be blocked by other objects in the plot, such as tree crowns or other stems. Consequently, cases exist where stems standing closer to the scanner reflect less laser points than those standing farther away from the scanner. Therefore, point number corresponds directly to the visibility conditions and should provide a better description of visibility in comparison with the distance criterion.

Table 8 summarizes the accuracy of the plot-level DBH measurements utilizing single-scan and multi-single-scan approaches, as reported in previous references. When the single-scan approach was utilized, the bias was between −1.6 cm and 1.58 cm, and the RMSE of DBH estimate was between 1.8 cm and 7.0 cm. In this study, the bias and RMSE of DBH estimate were 1.11 cm and 5.06 cm, respectively, when utilizing the multipass-corridor-mapping method and PLS; these results are close to those obtained using the single-scan approach. Overall, these results indicate that the PLS provides an alternative mapping technique for plot-level forest field measurements with substantially reduced occlusion effects.

It is worth noting that there are differences between DBH measurement methods [36]. For example, caliper measures a stem diameter in one direction, diameter tape gives an average of all diameters over all directions and PLS provides a fitted diameter whose direction is determined by the captured 3-D points. Therefore, variations inherently exist between DBHs from tape measurements in the field and PLS estimates, which can be on the order of several cm depending on cross-section eccentricity of the stems, as shown in [37]. In this experiment, the PLS-measured DBH is compared to reference DBH that was calculated from the tape-measured perimeter. The evaluation does not consider the variation between tape- and PLS-measurement and assumes that the DBH-estimate error is only due to the PLS measurement, which gives an exaggerated RMSE.

The DBH estimate of the multi-single-scan approach is more accurate than the results obtained using multipass-corridor-mapping. The multipass-corridor-mapping method estimates stem attributes in one path where the visibility of a tree stem is supposed to be the best. This mechanism is very close to the multi-single-scan approach. Therefore, different estimate results most likely came from the test data.

Different forest contexts, such as different species, varying amounts of ground vegetation and different developmental classes, influence the estimate accuracy of tree attributes. For example, the experiment plot in this study has a low stem density but contains many young trees in undergrowth that make the DBH estimate challenging. However, the main influence is expected to be the different characteristics of the laser point clouds collected by static TLS and PLS.

In the PLS, the point cloud was collected using a mobile system that included a scanner and a navigation unit wore by an operator. The point cloud collected by PLS was less accurate in comparison with the static TLS because navigation errors lead to inaccurate position estimates and these errors propagated in the laser point cloud.

**Figure 6.**
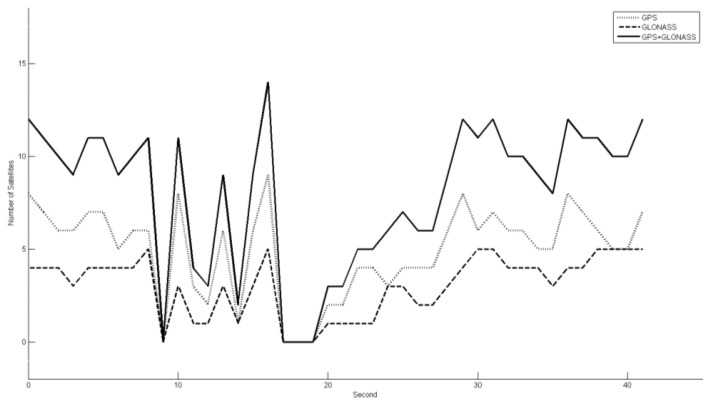
The number of GPS and GLONASS satellites tracked over time, individually and together.

Satellite signals under forest canopy can be weak and easily interrupted. Figure 6 shows the number of GPS and GLONASS satellites, individually and together, tracked by the GNSS receiver in approximately 40 s during the field measurements. As shown in the figure, multi-constellation GNSS improves the system performance in forested environments in comparison with a single-constellation GPS or GLONASS system. However, there are still cases of severe or complete signal loss. Therefore, location accuracy is typically worse in forests than in open areas.

The lack of proper satellite visibility leads to less accurate platform positions and also undermines the heading angle estimates. For example, a 0.05° heading error yields a 2 cm location measurement error at 25 m distances from the scanner. These errors are reflected finally in the georeferenced point cloud because the laser point cloud is calculated with the GNSS information. Consequently, the accuracy of PLS laser points is lower than static TLS measurements.

The RMSE of the DBH estimate was 5.06 cm in this study when utilizing the multipass-corridor-mapping approach and PLS data and it was 0.74–2.41 cm when utilizing the multi-single-scan approach and TLS data. The less accurate results from PLS data showed the positioning errors propagate to the point cloud data.

**Figure 7.**
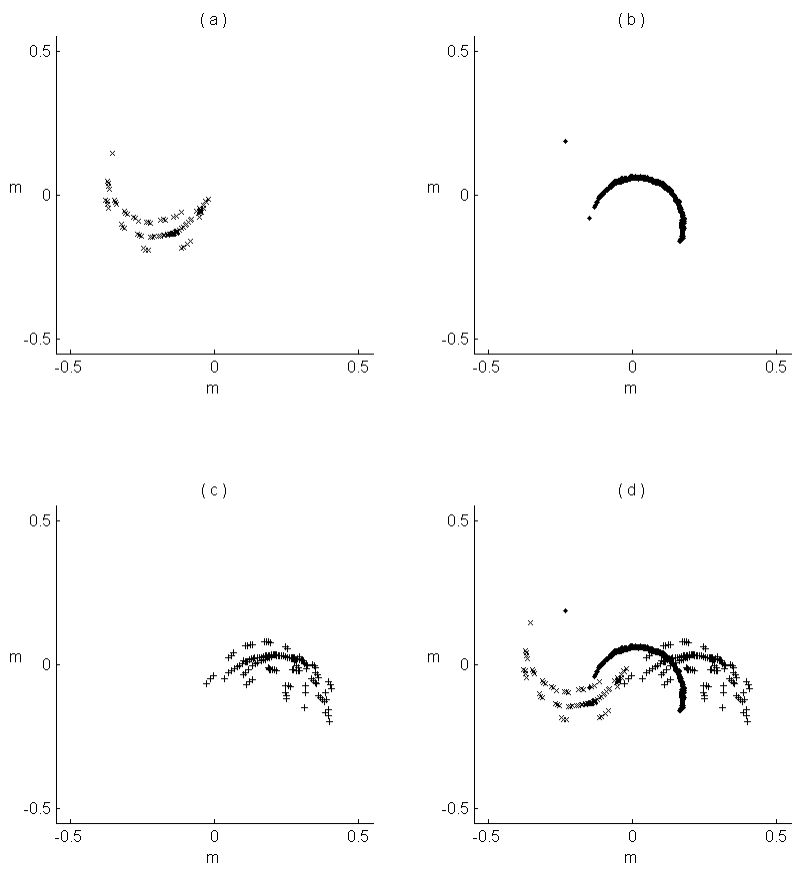
(**a**–**c**) The PLS points of a tree stem in a slice in three individual mapping paths and (**d**) in the merged data.

The multipass-corridor-mapping approach estimates the DBH of a tree stem in the path where the visibility of the tree is the best, so the point cloud includes single-path positioning errors. When utilizing the merged PLS point cloud, the RMSE of the DBH estimate was 8.45 cm, which is less accurate than the estimate utilizing the multipass-corridor-mapping approach. This result indicates merged PLS data include positioning errors caused by combining data from several paths, which makes it less accurate. Figure 7 shows the PLS points of a tree stem in a 5-cm-thick slice, in three individual mapping paths (a), (b) and (c) and in the merged data (d).

As shown in the Figure 7, the accuracy of the point cloud data from the PLS is largely dependent on the positioning errors in the GNSS system. The results of DBH estimate also indicate that the multipass-corridor-mapping approach, which utilizes a decision-level merging strategy, is an efficient method when PLS data are employed.

In general, the PLS provides a new measurement principle to document trees and other characteristics of forest plots. The results from the first experiment show that the PLS has a clear potential to be developed into an effective, convenient and practical tool for forest inventory and management purposes.

In addition to collecting tree and plot level data for forest inventories, the system can be used to objectively analyze the extent of canopy gaps, measure LAI from large areas and document and visualize forest routes feasible for recreation, hiking and berry and mushroom picking. The transmissivity of forest canopies can also be studied from the ground with the use of the PLS. In the application of ALS-based forest measurement, the transmissivity should be understood before studying terrain information under canopy covers. Currently, there is a lack of detailed information regarding the maximum effective measurement angle where the laser pulse still transmits through the canopy and back.

Harvester operations can also be improved by utilizing the PLS systems. Visiting forest areas before harvesting could provide preliminary information that is useful in the bucking process during harvesting. For example, a local stem curve allometric model created from point cloud data could optimize the bucking process and selection of trees to be cut. Because the system does not require professional knowledge of forests or laser scanners, this information can also be provided by the land owner prior to timber trading.

There are still several areas where improvements are needed. The technological disadvantages of the PLS need to be further studied. One clear challenge is the problem with positioning and orientation in dense forests. The results in this study showed that the estimated accuracy of tree features, such as DBH, is directly dependent on positional accuracies, as shown by the different accuracies of DBH estimates between using multipass-corridor-mapping and multi-single-scan approaches.

The accuracy of PLS (also MLS) is similar to that of ALS, which is based on the accuracy of the GNSS and IMU. When the visibility of GNSS satellites is poor, the quality of the applied IMU determines the system's capability to manage such challenging situations and determines the overall positioning performance. High relative accuracy is needed to attain a point cloud of trees with less than 1–2 cm deviations. In dense forest, positioning accuracy resulting from the use of GNSS and IMU is inadequate. Data correction methods should be developed to improve positioning reliability in PLS. In addition, multi-sensoral positioning techniques should be investigated.

In addition to the navigation components, other features in forested areas can also introduce positioning errors, such as the terrain and abrupt movements of the PLS system, which directly affect the point distribution on the object surfaces. The test area in this study is on a hill slope with understory and bare rocks. The operator may make more sudden movements in this type of terrain in comparison with flat terrain. The influence of terrain or movements needs further investigation. Additional discussion is also needed about methodologically minimizing such influences that are introduced by environmental features.

The results in this study showed that PLS data have clearly different characteristics in comparison with conventional, static TLS data. The positioning errors due to the position system produce clear variations in the PLS data. Therefore, it is expected that TLS processing methods cannot necessarily be directly applied to PLS data. Further research is needed to develop new processing methods for PLS point clouds.

In forest mapping, the use of PLS is not limited to circular or rectangular plots that are currently in wide use. The visible area beside a single strip may serve as a forest plot in the future. For example, a 20–50 m long and 6–20 m wide strip may be used as a plot, especially when ALS data are also utilized. Areas corresponding to visible trees will then to be utilized as the effective plot area. This possibility needs to be further explored and verified for feasibility and validity.

## 6. Conclusion

Advancements in sensor technology, system development and data analysis formulate the concept of the professional-quality personal laser scanning (PLS) system. This paper details the first experimental results from a PLS field test and demonstrated the applicability of such a system in a forested area for collecting tree attributes. The new PLS system has great potential for utilization in various forest ecosystem services as well as in other areas, such as cities and built environments. The multipass-corridor-mapping provides an effective method of processing data collected utilizing a PLS system.

Because of positioning errors, quality of PLS data can be expected to be lower in comparison with static data acquisition instruments such as terrestrial laser scanning, especially in forests with dense tree crowns that blocks GNSS signals. Currently, the RMSE of PLS DBH estimate is about 5 cm. This error is large and may introduce substantial bias when the PLS DBH estimates are utilized in estimating other tree features such as volume and biomass. High quality data are one of the key factors of applying PLS in forest mapping and ecosystem services. Further development is needed for data correction methods and multi-sensoral positioning. Additionally, further research is needed to explore the mapping accuracy in different types of forests and the influences of environmental factors, such as forests with rough and/or steep terrain and with a lot of branches and/or understory. The possibilities of applying the new plot concept (visible area besides a single strip) should also be further discussed.

In general, PLS is expected to be a very practical and convenient instrument to make the tree inventory and to document other tree characteristics in forested areas where the quality of PLS data matches the requirement of the application. The technological disadvantages and the new application possibilities of PLS need to be further studied.

## Figures and Tables

**Table 1.** Specifications of the navigation system (NovAtel SPAN GNSS-IMU).

**Unit**	**Features**	**Specifications**
NovAtel Flexpak6 GNSS receiver and GPS-702GG antenna a	Frequency	GPS L1 L2 L2C GLONASS L1 L2
	Horizontal position accuracy (RMS) RTK	1 cm + 1 ppm
NovAtel UIMU-LCI Fiber optic gyro IMU b	Output rate Acceleration accuracy (RMS) Gyro input range Gyro rate bias Gyro rate scale factor Angular random walk Accelerometer range Accelerometer bias	200 Hz 0.004 m/s^2^ ±800 deg/s <1.0 deg/h 100 ppm <0.05 deg/√*h* ±40 g <1.0 mg

a www.novatel.com/assets/Documents/Papers/FlexPak6.pdf;

b http://www.novatel.com/assets/Documents/Papers/IMU-LCI.pdf.

**Table 2.** Specifications of the laser scanner (FARO Focus3D 120 a).

**Features**	**Specifications**
Maximum range	153 m
Vertical field of view	305 degrees
Ranging error	±2 mm @ 25 m
Wavelength	905 nm
Beam divergence	0.19 mrad, 3 mm at exit
Scan frequency	3–97 Hz
Point repetition frequency	122–976 kHz
Angular resolution	0.02–5.0 mrad

a FARO Focus3D Features, Benefits & Technical Specifications, www.faro.com.

**Table 3.** Descriptive statistics of the trees in the study area.

		**Species**			

		**Scots Pine**	**Norway Spruce**	**Deciduous**	**Sum**
	(No. of stems)	35	1	10	46
	(%)	76.09	2.17	21.74	100
		min.	max.	mean	std. dev.
DBH	(cm)	10.22	51.25	31.86	11.48

**Table 4.** Stem mapping accuracy utilizing the multipass-corridor-mapping method and PLS data.

	**Reference**	**Mapped**	**Omission**	**Commission**
Number (stem)	46	38	8	11
Percentage (%)	100	82.6	17.4	23.9

**Table 5.** The accuracy of the DBH and position estimate utilizing the multipass-corridor-mapping method and PLS data.

	**Bias**	**Bias%**	**RMSE**	**RMSE%**
DBH (cm)	1.11	3.20	5.06	14.63
Position (m)	0.34	\	0.38	\

**Table 6.** Stem mapping accuracy utilizing the merged PLS data.

	**Reference**	**Mapped**	**Omission**	**Commission**
Number (stem)	46	34	12	10
Percentage (%)	100	73.9	26.1	21.7

**Table 7.** The accuracy of the DBH and position estimates utilizing the merged PLS data.

	**Bias**	**Bias%**	**RMSE**	**RMSE%**
DBH (cm)	4.37	12.25	8.45	23.71
Position (m)	0.36	\	0.39	\

**Table 8.** Summary of the plot-level DBH estimate utilizing single-scan and multi-single-scan approaches reported in the previous references.

	**Method**	**Number**	**Plot size**	**Result**		

				**Density (stems/m^2^)**	**Bias DBH (cm)**	**RMSE DBH (cm)**
Maas *et al.*, 2008 [16]	single-scan	3	15 m radius	0.0212–0.041	−0.67–1.58	1.80–3.25
Brolly and Kiraly, 2009 [17]*	single-scan	1	30 m radius	0.0753	−1.6–0.5	3.4–7.0
Lindberg et al., 2012 [35]**	single-scan	6	80 × 80 m	0.0519–0.0663	0.16**	3.8**
Liang and Hyyppä, 2013 [34]	multi-single-scan	5	10 m radius	0.0605–0.121	−0.18–0.76	0.74–2.41

* Three detection methods were discussed;

** The results were reported for all detected trees in all test plots, thus this number can be understood as an average value.
